# Methane Emission by Camelids

**DOI:** 10.1371/journal.pone.0094363

**Published:** 2014-04-09

**Authors:** Marie T. Dittmann, Ullrich Runge, Richard A. Lang, Dario Moser, Cordula Galeffi, Michael Kreuzer, Marcus Clauss

**Affiliations:** 1 Clinic for Zoo Animals, Exotic Pets and Wildlife, Vetsuisse Faculty Zurich, University of Zurich, Zurich, Switzerland; 2 ETH Zurich, Institute for Agricultural Sciences, Zurich, Switzerland; 3 Kamelhof Olmerswil, Neukirch/Thur, Switzerland; 4 Cochranton Veterinary Hospital, Cochranton, Pennsylvania, United States of America; 5 Zoological Institute, University of Basel, Basel, Switzerland; 6 Zurich Zoo, Zurich, Switzerland; The University of Wollongong, Australia

## Abstract

Methane emissions from ruminant livestock have been intensively studied in order to reduce contribution to the greenhouse effect. Ruminants were found to produce more enteric methane than other mammalian herbivores. As camelids share some features of their digestive anatomy and physiology with ruminants, it has been proposed that they produce similar amounts of methane per unit of body mass. This is of special relevance for countrywide greenhouse gas budgets of countries that harbor large populations of camelids like Australia. However, hardly any quantitative methane emission measurements have been performed in camelids. In order to fill this gap, we carried out respiration chamber measurements with three camelid species (*Vicugna pacos*, *Lama glama*, *Camelus bactrianus*; n = 16 in total), all kept on a diet consisting of food produced from alfalfa only. The camelids produced less methane expressed on the basis of body mass (0.32±0.11 L kg^−1^ d^−1^) when compared to literature data on domestic ruminants fed on roughage diets (0.58±0.16 L kg^−1^ d^−1^). However, there was no significant difference between the two suborders when methane emission was expressed on the basis of digestible neutral detergent fiber intake (92.7±33.9 L kg^−1^ in camelids vs. 86.2±12.1 L kg^−1^ in ruminants). This implies that the pathways of methanogenesis forming part of the microbial digestion of fiber in the foregut are similar between the groups, and that the lower methane emission of camelids can be explained by their generally lower relative food intake. Our results suggest that the methane emission of Australia's feral camels corresponds only to 1 to 2% of the methane amount produced by the countries' domestic ruminants and that calculations of greenhouse gas budgets of countries with large camelid populations based on equations developed for ruminants are generally overestimating the actual levels.

## Introduction

The quantification and abatement of methane (CH_4_) emissions from domestic ruminants have received major attention from the scientific community during the last decades [1]–[4]. Ruminants digest fibrous carbohydrates by microbial fermentation of plant material in their gastrointestinal tract [5]. One of the side products of this fermentation process is CH_4_, a greenhouse gas (GHG) that also represents a loss of energy to the host animal [1].

Among mammals, ruminants (*Ruminantia*) produce the highest amounts of CH_4_ in relation to body mass, yet explanations for this finding remain speculative [6]. Some of the features that characterize ruminants, like the ability to ruminate and a chambered foregut that enables the sorting of food particles according to size, are shared with another artiodactyl suborder, the camelids (*Tylopoda*) [7]–[10]. Given these similarities in digestive anatomy and physiology, it has been assumed that camelids produce similar amounts of CH_4_ as ruminants when compared at the same body mass range [6], [11], [12] and are thus responsible for the release of significant amounts of this GHG. However, despite the similarities to the ruminant digestive anatomy and physiology, there are some important differences between the two suborders:

1. The camelid foregut can be separated into three compartments [7], [8]. The first two compartments (C1 and C2) represent a fermentation chamber similar to the reticulorumen of ruminants. The last elongated tubular compartment (C3) shows similarities to the abomasum of ruminants [7]. Despite structural similarities with the ruminant foregut, the camelid compartments cannot be considered as direct homologues [8].
2. Camelids have a lower food intake compared to ruminants [13], which corresponds to their lower energy requirements [14]. This can be interpreted as an adaptation to environments with low resource availability.
3. Food particles are retained longer in the camelid foregut than in the ruminant foregut [15]. This could be explained by the lower intake of food, and results in a longer time of fermentation, which is a prerequisite for effective fiber digestion. It has also been suggested that longer particle retention is achieved by the delayed start of rumination after feeding compared to ruminants [10].
4. The mechanism of particle sorting in the forestomach appears to be similarly density-dependent in camelids and ruminants [9]. However, some proportions of large particles are found in the last camelid forestomach compartment (C3), where no further breakdown of particles takes place. Large particles are not found in the distal digestive tract or feces, these large particles need to be returned to the C1/C2 compartments from which they can be re-submitted to further size reduction via rumination [9]. This particularity of retaining very large particles in the last compartment could represent a limitation for food intake.
5. Camelids were reported to have a higher efficiency in dry matter and fiber digestion than ruminants [16]–[19]. This is probably achieved by a longer retention of particles and not by different fermentation pathways, as composition of the microbial community in the camel gut resembles the one in ruminants [20], [21]. The longer particle retention and the consequently longer exposition to microbial fermentation could result in a higher CH_4_ production per unit food ingested when compared to ruminants.

Taken together, there are notable differences in the anatomy and physiology of the digestive tract between camelids and ruminants, which may influence microbial CH_4_ production. Relatively little is known about CH_4_ emission by camelids. Hackstein and Van Alen [4] detected methanogenesis in the feces of Bactrian camels (*Camelus bactrianus*), alpacas (*Vicugna pacos*) and guanacos (*Lama guanaicoe*). A study on methanogenic archeae in the alpaca foregut revealed the presence of *Methanobrevibacter* strains, which are the most common methanogens in ruminants, at similar densities as reported for ruminants [22]. The occurrence of production of enteric CH_4_ was confirmed for dromedaries (*Camelus dromedarius*) [23], [24], llamas (*Lama glama*) [25], [26] and alpacas [27]–[30].

Including livestock emissions into global GHG surveys revealed that enteric fermentation, mostly of ruminants, contributes approximately 20 to 25% to the observed increase in atmospheric CH_4_ [31]. Such estimates are generally developed based on equations for ruminants and animal population sizes of the respective countries [12]. Therefore, specific data for CH_4_ emission from camelids are interesting for calculating GHG budgets of countries that harbor large populations of camelids like several African and South American countries as well as Australia [32], [33].

To fill this gap of knowledge, we measured CH_4_ emission in three camelid species and compared them with literature data from ruminants. Our hypotheses were that (i) camelids produce less CH_4_ than ruminants per kg of body mass (BM) because it is known that their food intake per capita is lower than that of ruminants of similar size [13], [14]. Given the longer time of particle retention camels [15], which results in a longer time available for fermentation of the digesta and, thus, in a higher nutrient digestibility [16]–[18], (ii) CH_4_ production per unit food ingested was expected to be higher in camelids than in ruminants. The same was expected for methane expressed as percentage of digestible energy intake (DEI) as a higher digestibility might result in a production of higher amounts of CO_2_ and H_2_, the substrates for CH_4_. This has already been shown in sheep [34] (iii). As fiber is the main substrate for methanogens [35], CH_4_ emission should be determined especially by the amount of digestible fiber ingested by the animal. Despite the differences in digestive anatomy and physiology between ruminants and camelids, we further assumed that the process of fiber digestion itself and the pathways of methanogenesis are similar in both groups and that, therefore, (iv) camels produce the same amount of CH_4_ when expressed on a basis of digestible neutral detergent fiber intake (dNDFI).

## Methods

### Ethics statement

Animal trials in this study were approved by the Kantonales Veterinäramt Zürich, Switzerland, and took place under the Swiss Cantonal Animal Experiment Licence no. 142/2011.

### Study species

Measurements were carried out on three camelid species that were chosen to cover a range of body mass corresponding that of domestic ruminants. The smaller two species, alpacas and llamas, belong to the SAC. Despite uncertainties about their taxonomic affiliations, llamas and alpacas are considered to be the domesticated forms of the guanaco (*Lama guanicoe*) and vicugna (*Vicugna vicugna*), originating in the Andean region [36]. The third species selected was the Bactrian camel, the largest member of this suborder, which was originally distributed over the Asian continent, while nowadays only few remaining free-ranging individuals roam small desert areas in Mongolia and China [37].

### Respiration measurements

Five alpacas kept at Zurich zoo, and six llamas and five Bactrian camels kept on a private camel farm in Switzerland were separated and kept in individual pens. Animals had access to a diet consisting of alfalfa hay provided at *ad libitum* access and a limited amount of alfalfa pellets (Table 1). Alfalfa pellets made up 53±10, 33±6 and 21±2% of DMI in alpacas, llamas and Bactrian camels respectively. They had unrestricted access to water. Details on the experimental animals are given in **Table 2**.

**Table 1:** Nutrient composition of the diet items used in the present study (in g/kg dry matter and MJ/kg dry matter for GE).

Diet item	Species	TA	CP	EE	CF	NDF	ADF	ADL	GE
Alfalfa hay	Alpaca	8.3	14.8	1.0	37.8	58.5	38.5	8.4	18.3
	Llama	9.6	13.3	0.9	40.0	59.2	44.6	9.2	18.1
	Bactrian camel	9.6	16.3	1.0	41.4	56.2	45.6	9.9	17.9
Alfalfa pellets*	All camelids	11.9	16.6	1.6	26.6	40.8	33.3	7.9	18.3

TA total ash, CP crude protein, EE ether extracts, CF crude fiber, NDF neutral detergent fiber, ADF acid detergent fiber, ADL acid detergent lignin, GE gross energy.

*No. 2805, Provimi Kliba SA, Kaiseraugst, Switzerland.

**Table 2:** Animals used in the present study and individual data on body mass, food and digestible energy intake, and methane production.

Species	Age	Sex	BM	DMI	NDFI	dNDFI	DEI	CH_4_						
								Own measurements				Estimate based on Kirchgessner et al. [51] *	Ratio measured to estimated	Ratio to CO_2_ produced
	y		kg	kg d^−1^	kg d^−1^	kg d^−1^	MJ d^−1^	L d^−1^	L kg^−1^ DMI	% DEI	L kg^−1^ dNDFI	L d^−1^		
*Vicugna pacos*	2	F	50	0.9	0.4	0.2	9.6	13.1	14.7	5.2	9.4	64.2	0.20	0.084
*Vicugna pacos*	4	F	53	1.3	0.6	0.4	15.3	33.2	26.1	8.4	23.8	74.5	0.45	0.100
*Vicugna pacos*	3	F	64	1.0	0.4	0.1	8.6	27.1	27.7	12.2	19.4	66.0	0.41	0.082
*Vicugna pacos*	15	F	71	1.4	0.6	0.3	16.0	22.1	15.9	5.3	15.8	75.7	0.29	0.102
*Vicugna pacos*	10	M	79	1.2	0.5	0.2	12.1	17.3	15.0	5.5	12.4	70.0	0.25	0.081
*Lama glama*	4	F	110	2.3	1.1	0.6	24.2	51.1	22.3	8.1	36.6	99.1	0.52	0.082
*Lama glama*	7	M	140	2.2	1.0	0.5	20.4	41.1	19.1	7.8	29.4	95.2	0.43	0.068
*Lama glama*	4	F	140	2.4	1.2	0.6	24.1	48.1	19.9	7.7	34.4	102.0	0.47	0.074
*Lama glama*	11	M	150	1.9	0.9	0.3	15.6	47.5	24.6	11.7	34.0	89.2	0.53	0.075
*Lama glama*	5	F	160	2.5	1.3	0.6	24.2	46.5	18.3	7.4	33.3	105.7	0.44	0.084
*Lama glama*	7	M	190	3.4	1.8	0.9	34.6	71.0	20.8	7.9	50.8	128.7	0.55	0.088
*Camelus bactrianus*	13	M	590	9.4	4.8	2.2	89.0	154.2	16.4	6.7	110.4	284.6	0.54	0.080
*Camelus bactrianus*	5	M	600	9.3	4.8	2.0	84.6	185.5	20.0	8.5	132.8	282.0	0.66	0.086
*Camelus bactrianus*	6	M	640	8.6	4.4	1.8	80.4	117.8	13.8	5.6	84.3	262.1	0.45	0.069
*Camelus bactrianus*	7	M	700	7.5	3.9	1.5	65.0	131.3	17.5	7.8	94.0	233.7	0.56	0.084
*Camelus bactrianus*	7	F	760	7.9	4.2	1.7	73.9	154.5	19.5	8.1	110.6	244.6	0.63	0.070

BM body mass, DMI dry matter intake, NDFI neutral detergent fiber intake, dNDFI digestible neutral detergent fiber intake, DEI digestible energy intake, y years, F female, M male.

*Estimated based °n a regression equation developed from domestic ruminants that uses information about diet nutrient composition (see Methods).

**Table 3:** Data on average CH_4_ production of camelids and ruminants obtained by respiration measurements sorted by animal size.

Group		Mean BM (SD); *n*	Mean CH_4_ production (SD); *n*			
		kg	L d^−1^ kg BM^−1^	L kg DMI^−1^	% DEI	L kg dNDFI^−1^
All	Camelids	259 (±260); *18*	0.32 (±0.11); *18*	20.1 (±4.4); *18*	8.0 (±2.3); *17*	92.7 (±33.9); *17*
	Ruminants	161 (±211); *48*	0.58 (±0.16); *48*	28.1 (±6.0); *34*	11.7 (±2.8); *23*	86.2 (±12.1); *10*
Small	South American camelids	106 (±46); *13*	0.35 (±0.10); *13*	21.2 (±4.6); *13*	8.3 (±2.6); *12*	97.4 (±39.1); *12*
	Sheep and goats	53 (±22); *37*	0.55 (±0.17); *37*	26.4 (±5.1); *24*	11.6 (±2.4); *17*	86.8 (±11.5); *6*
Large	Bactrian camels	658 (±72); *5*	0.23 (±0.05); *5*	17.4 (±2.5); *5*	7.3 (±1.1); *5*	81.5 (±12.7); *5*
	Cattle	525 (±140); *11*	0.66 (±0.10); *11*	32.0 (±6.6); *10*	12.0 (±4.2); *6*	85.3 (±14.9); *4*

Note that sample size corresponds to the number of individuals used for measurements in the present study but to means from different publications for ruminants. Data sources are Table 2 for the present study and in Table S1 for literature data. BM body mass, DMI dry matter intake, DEI digestible energy intake, dNDFI digestible neutral detergent fiber intake.

In order to determine DMI, digestible NDF intake (dNDFI), and DEI, food supply, refusal and feces amounts were weighed daily during one week before the CH_4_ measurements, and representative samples were taken. After the animals were weighed on a mobile scale (alpacas) or a truck scale (llamas, Bactrian camels), they were put separately into the respiration chambers for one 24 h period. For the alpacas, a transport box of a size of 1.9×0.7×1.3 m was used as chamber, for the llamas and the Bactrian camels a part of a building was separated by wooden panels to build boxes of 2.9×1.6×2.4 m and 4.5×2.9×2.4 m, respectively. To prevent air leaks, the chambers were sealed with plastic foil (Building and covering film, 0.2 mm, Folag AG Folienwerke, Sempbach, CH), silicone and tape. In the chambers the animals also had free access to alfalfa hay and water, and a limited amount of alfalfa pellets.

Chambers were fitted with a series of air inlets at the bottom and a series of air outlets at the top of the chamber, that were connected to an air pump (Flowkit 500, Sable Systems, Las Vegas, USA), which ensured a slight under-pressure in the chamber and constant flow rates of 48 to 72 L min^−1^ for alpacas, 116 to 148 L min^−1^ for llamas and 362 to 460 L min^−1^ for Bactrian camels, respectively. Levels of CH_4_, oxygen, carbon dioxide, water vapor pressure and barometric pressure were measured by gas analyzers (MA-10 and Turbofox, Sable Systems) from ambient air and air sampled from the chambers at alternating intervals of 90 s each. Wash out times for the system ranged at 10 seconds and readings were corrected for this time lag. For data analysis, we only used measurements recorded after gas levels in the chamber had reached a stable plateau, which occurred 60 to 150 min after the animals had been placed in the boxes. Animals were under constant monitoring throughout the measurements.

Gas analyzers were calibrated prior to each measurement by using pure nitrogen and a calibration gas (PanGas, 19.91% O_2_, 0.51% CO_2_, 0.49% CH_4_ dissolved in nitrogen). Data obtained by the respiratory system were analyzed with the software ExpeData (Sable Systems) where the mean CH_4_ concentration was calculated and corrected for CH_4_ concentration in ambient air, partial pressures of oxygen, carbon dioxide and water vapor as well as barometric pressure.

### Sample analysis

Nutrient contents of the samples from food, refusals and feces were analyzed using standard procedures [38], [39]. All samples were oven-dried at 65°C and ground to 0.75 mm with a mill (Retsch GmbH, Haan, Germany). Samples were analyzed for dry matter content by drying at 103 °C to constant weight. Gross energy (GE) was determined by bomb calorimetry (IKA-Calorimeter C4000, Ika, Stauffen, Germany). Total ash (TA) was analyzed using a muffle furnace [40]. For determinations of nitrogen by the Dumas method, an Elementar rapid N III Analyzer (Elementar Analysensysteme, Hanau, Germany) was used. Crude protein (CP) was calculated as 6.25×N [41]. Crude fiber (CF), NDF (after treatment with α-amylase), ADF and ADL contents were determined using the Fibertec System M (Tecator, 1020 Hot Extraction, Flawil, Switzerland; AOAC 962.09). The fiber data were corrected for ash content. Ether extract (EE) was analyzed with a Soxhlet extractor system (Extraktionsapparatur B-811, Büchi, Flawil, Switzerland; AOAC 963.15). Nitrogen free extract (NfE) was calculated as 100 – TA (%) – CP (%) – EE (%) – CF (%).

Nutrient data were used to predict the expected amount of CH_4_ produced by domestic cattle on the corresponding diet using the equation of Kirchgessner et al. [42]: CH_4_ (g d^−1^) = 63 + 80×CF (kg d^−1^) + 11×NfE (kg d^−1^) + 19×CP (kg d^−1^) – 195×EE (kg d^−1^).

### Literature data

Apart from the scarce CH_4_ data on camelids, where the animals had received a roughage-only diet, literature data were collated from the three most common domestic ruminant species, i.e. cattle (*Bos taurus* and *Bos indicus*), sheep (*Ovis aries*) and goat (*Capra hircus*). Only measurements that could be related to BM, DMI, and, if possible, DEI and dNDFI (see Table S1 for data and sources) were used. Because of the differences in the level of detail reported in the various literature sources, the corresponding datasets differed distinctively in sample size. We only selected data from animals that were fed on roughage to allow comparison to the data obtained from our respiration measurements, and to broadly exclude the effect of diet (as in roughage vs. concentrate feeds). Only data obtained by measurements in respiration chambers were used.

### Statistical evaluation

In order to investigate how much CH_4_ camelids produce in relation to domestic ruminants, we applied general linear models (GLM) with CH_4_ production (L per day, L per kg DMI, % of DEI, and L per kg dNDFI) as the dependent variable, and body mass, suborder (SO, ruminant or camelid) and, when available for the majority of the data points within a dataset, NDF content of the diet as fixed effects. The interaction between BM and SO was also included as fixed effect, but was removed from the model when it was not statistically significant. In the case of a significant interaction, we performed separate analyses on two subgroups of different body mass ranges consisting of alpaca and llama (SAC) in comparison to sheep and goats (subgroup: small) and Bactrian camels in comparison to cattle (subgroup: large) by applying either GLMs or, when there was no significant effect of BM, Wilcoxon ranked sum tests. Analyses were carried out with ln-transformed values for BM and the dependent variables.

In addition to that, we tested whether the data obtained by our camelid measurements were actually in the range that would be expected for ruminants on the experimental diet by subjecting our data to the equation of Kirchgessner et al. [42] and by applying a Mann-Whitney-U-test to compare the correspondingly estimated data with the measured data. In order to test whether CH_4_ emissions correlate with indicators of energy metabolism, we incorporated emissions of CH_4_ and CO_2_ (L d^−1^) into a linear model and calculated the average ratio of CH_4_:CO_2_ to compare it to ruminant values from the literature. All statistical tests were carried out with R 3.0.2 [43] and significance levels were set to α = 0.05, with values between 0.05 and 0.10 considered as trends.

## Results

**Figure 1:**
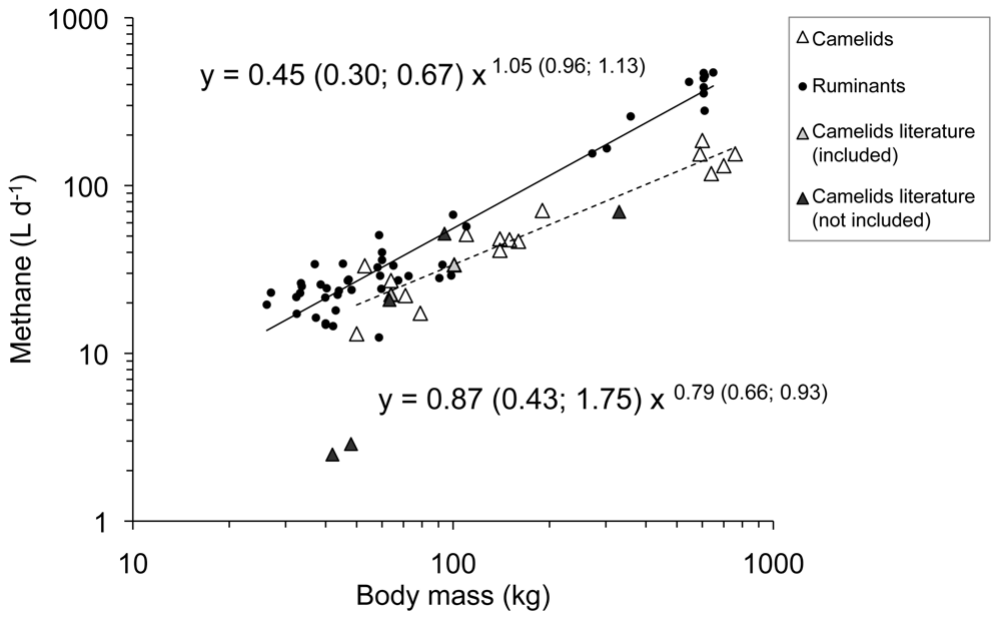
Methane emission in L d^−1^ of domestic ruminants (literature data) and camelids (own measurements, literature data included in the regression analysis and literature data not included due to differences in methodology) in relation to body mass. 95% confidence intervals of the regression lines are given in brackets. R^2^ values of the regression lines are 0.93 for ruminants and 0.91 for camelids. For data sources see Table S1.

The dataset on CH_4_ in L d^−1^ contained 18 camelid and 48 ruminant data points. In this dataset, the interaction of body mass and suborder (BM×SO) was significant (F_1,65_ = 8.40; P = 0.005), which is why the two animal subgroups (small and large) were tested separately. For the smaller animals, there was no significance for the interaction of BM×SO but an effect of BM (F_1,49_ = 40.80; P<0.001) and a trend (F_1,49_ = 3.42; P = 0.071) towards lower CH_4_ emission from the SAC compared to the smaller ruminants. Within the larger animals, camels produced significantly less CH_4_ per day than cattle (W = 2; P = 0.002) (Figure 1; Table 3 for means). The dietary NDF contents were available for 17 camelid and 18 ruminant data points in this dataset. The analysis of this reduced dataset revealed that the NDF content of the diet was no significant covariable (F_1,34_<0.001; P = 0.99).

The dataset on CH_4_ in L per kg DMI contained 18 camelid and 34 ruminant data points. In this dataset, there was an interaction of BM×SO (F_1,51_ = 5.58; P = 0.022). Testing the two subgroups separately revealed no effect of BM in SAC (F_1,39_<0.001; P = 0.987), and lower CH_4_ emissions per kg DMI in both large and small camelids compared to ruminants (small: W = 73; P = 0.005; large: W = 0; P<0.001). Dietary NDF contents in this dataset were available for 17 camelid and 18 ruminant data points. In this reduced dataset, NDF content of the diet was a significant covariable (F_1,34_ = 2.67; P = 0.012), suggesting that in the case of expressing CH_4_ per DMI the difference between the suborders is due to the different fiber levels of the forages used in the experiments evaluated. In this context, the NDF content in the diet was on average higher in ruminants (59%) than in camelids (50%) (W = 71; P = 0.007).

The dataset on CH_4_ in % DEI contained 17 camelid and 23 ruminant data points. In this dataset, there was no BM×SO interaction (F_1,39_ =  0.24; P = 0.625) and no effect of BM (F_1,39_ = 0.05; P = 0.827). Methane emissions in % DEI were lower in camelids than in ruminants (W = 59; P<0.001). Dietary NDF contents in this dataset were available for 17 camelid and 12 ruminant data points and proved to be a significant covariable (F_1,28_ = 16.2; P<0.001). This again suggests that when expressing CH_4_ per DEI, any difference between animals is due to the fiber content of the forages used in the experiments. In this dataset, NDF content in the diet was on average 57% in ruminants and 50% in camelids (W = 152; P = 0.028).

**Figure 2:**
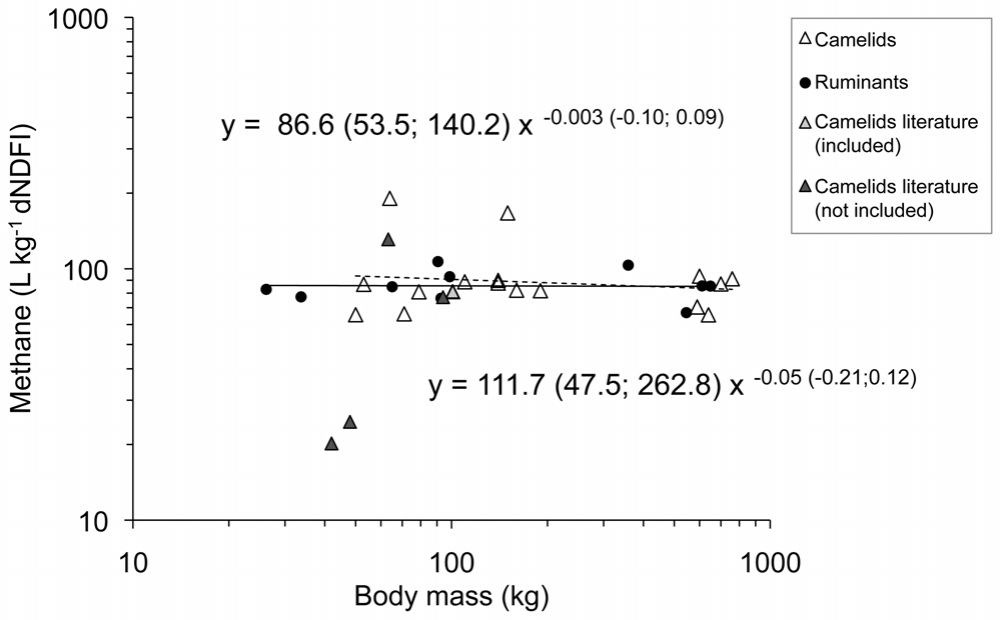
Methane emission in L per kg digestible neutral detergent fiber intake (dNDFI) of domestic ruminants (literature data) and camelids (own measurements, literature data included in the regression analysis and literature data not included due to differences in methodology) in relation to body mass. 95% confidence intervals of the regression lines are given in brackets. R^2^ values of the regression lines are 0.02 for ruminants and<0.001 for camelids. For data sources see Table S1.

The dataset on CH_4_ per kg dNDFI contained 17 camelid and 10 ruminant data points. In this dataset, there was no interaction of BM×SO (F_1,23_ = 0.19; P = 0.663) and no effects of BM (F_1,23_  =  0.47; P = 0.501) and NDF content (F_1,23_ = 0.01; P = 0.927). There were also no differences between ruminants and camelids (W = 83; P = 0.941) (Figure 2).

In order to test whether the sample of the domestic ruminants influenced the results, we repeated all analyses (for CH_4_ per kg BM, per kg DMI and in % DEI) using only the 10 data points for domestic ruminants for which data in CH_4_ per kg dNDFI were available. The outcome did not differ from the results based on the larger datasets.

The amount of CH_4_ measured from the Bactrian camels in this experiment on average amounted only to 46% of the CH_4_ production estimated from the equation derived from ruminant data [42] (U = 0; P<0.001). This was very similar to the difference found in absolute CH_4_ production in the larger animals, where Bactrian camels produced 47.5% of the level of CH_4_ production described for cattle in L d^−1^. The CH_4_ emission of the camelids correlated highly with CO_2_ emissions (R^2^ = 0.98; P<0.001) and the CH_4_:CO_2_ ratio was 0.082±0.010.

## Discussion

### Level of methane emissions by camelids

Only few comparable literature data on CH_4_ emissions by camelids are available for inclusion into the overall analysis. To the knowledge of the authors, no CH_4_ measurements have been obtained in Bactrian camels before. We are aware that limiting measurements to 24 h, as done for the animals in the present study, might be somewhat biased due to variation between days in physical condition, feeding behavior or stress of the animals remaining unaccounted for. However, literature data on CH_4_ measurements obtained in llamas [26] and alpacas [29] kept on a roughage-only diet were incorporated in our analysis and turned out to be in the range of the values measured in this study, indicating the reliability of data derived under similar conditions from respiration measurements. Besides these scarce data, some CH_4_ measurements in camelids have been published that were not obtained by chamber respirometry or not on a roughage-only diet [24], [25], [27], [28], [30]. Despite the different measurement conditions, these values are mostly consistent in magnitude with our measurements (Figure 1 and 2).

### Methane emissions by camelids in comparison to ruminants

Our evaluation demonstrated that camelids produce less CH_4_ than ruminants when expressed on a basis of BM, but that CH_4_ production does not differ when expressed on a basis of digestible NDF intake. The differences observed in CH_4_ production between suborders when expressed per unit of dry matter or digestible energy intake are most likely due to the disparity in average fiber contents of the forages fed in the studies evaluated to either camelids (lower in fiber content) or ruminants. Total CH_4_ production per day in camelids, expressed per kg body mass, were on average only 56% of that reported for ruminants. In contrast, when expressed per unit of dry matter intake, camelid CH_4_ production was on average 73% of that in ruminants, which mirrors the lower fiber content of the diet the camelids received compared to the ruminants. In contrast to our prediction, the putatively higher digestive efficiency of camelids did not lead to higher methane values when expressed per unit food intake.

The least biased variable to compare methanogenesis from nutrient digestion is the amount of CH_4_ produced per unit of NDF digested. Because, in ruminants, methane is formed from CO_2_ and H_2_, which are products of microbial fermentation of carbohydrates [44], [45], fiber is considered the major substrate for methanogenesis [35]. Analyzing CH_4_ produced per unit of NDF digested excludes other influences on digestive efficiency, such as different fermentation conditions or a different digesta passage rate. Indeed, from the present evaluation it is obvious that camelids produce as much CH_4_ per unit of digestible NDF as ruminants. This suggests that the pathways of methanogenesis via microbial fermentation might not differ between the two suborders. Differences between suborders in the amount of CH_4_ produced therefore reflect the amount of fiber the animal digested, which in turn is determined by the general intake level. The most likely explanation for the lower absolute CH_4_ production in camelids, therefore, is their generally lower metabolism associated with lower nutrient requirements and thus a lower food intake per unit of body mass [13], [14]. This can be assumed to reflect an adaptation to environments characterized by low resource availability. A low metabolism and intake is also indicated by a low CO_2_ production per unit of BM. Therefore a similar CH_4_:CO_2_ ratio can be expected in camelids and ruminants, which was actually the case. Levels reported for ruminants are ranging between 0.050 and 0.096 [46]–[49] compared to the average of 0.082 found in the camelids of the present study.

### Implications of the findings of low methane emissions by camelids

Methane production estimates for camelids, derived from an often-used equation developed for ruminants based on nutrient composition of the diet [42], were more than twice as high as the actually measured methane amounts. Therefore, this and similar equations do not seem appropriate to predict CH_4_ emissions of camelids. Even conventional estimates based on the IPCC [12] default equation based on Y_m_ (the ratio of CH_4_ energy to GE intake) are often applied incorrectly because the proportionately lower GE intake as a consequence of the camelids' lower food intake is not considered. This is important when calculating GHG budgets for countries that harbor large populations of camelids, such as in northeastern Africa, South America [32] or Australia [33]. In general, equations developed for livestock to estimate CH_4_ emissions from any non-domestic species have to be applied carefully and assessments should rather rely on specific measurements.

Numerous approaches have recently been considered to reduce the contribution of enteric CH_4_ from livestock to the greenhouse effect. Among others, the mitigation of emissions from introduced feral one-humped camels has been discussed in Australia, a country that harbors the fifth largest population of dromedaries in the world [50]. The increasing negative impacts of these non-endemic animals on the Australian ecosystem initiated the search for appropriate management solutions [51], [52]. Statements that camels emit large amounts of CH_4_ and thereby intensively contribute to the GH effect [53] promoted calls for large-scale culling of these animals. However, the assumptions made concerning methane emission from the camels were based on estimates following the IPCC guidelines for national GHG inventories [12], with their limited applicability for camelids. While there is little doubt that the culling of any herbivore will reduce GHG emissions, the quantity of that reduction must be balanced against the costs of the culling. Our data suggest that a 570 kg dromedary emits approximately 131 L CH_4_ d^−1^, i.e. less than half as much as cattle of a similar size (approx. 357 L d^−1^). This corresponds to an annual amount of 36 kg CH_4_ per camel, which is clearly below the 46 kg assumed by Gibbs and Johnson [54] for a camel of the same weight and the 58 kg assumed by Crutzen et al. [55]. In total, Australia harbors 28.4 million cattle, 75.7 million sheep [56] and 1 million feral dromedaries [33]. This is equivalent to an estimated annual amount of 4500 billion L CH_4_ emissions from the domestic ruminants and only 48 billion L produced by the dromedary population. Culling of all feral camels would thus have a similar effect as reducing the livestock ruminant population by 1 to 2%. However, other detrimental impacts caused by the feral camels on the Australian environment underline the continued importance of management strategies.

### Conclusions

Methane emission was measured from three camelid species, including, for the first time, Bactrian camels. Our findings indicate that, in absolute values, camelids produce clearly less CH_4_ than ruminants, and that this difference is most likely due to the generally reduced metabolism, food and (digestible) fiber intake of this group. Therefore, when calculating GHG budgets, equations developed for ruminants are not applicable for the estimation of CH_4_ emissions from camelids.

## Supporting Information

Table S1 **Dataset including the literature data used for statistical comparison of camelids and ruminants.**
(DOCX)
