# Supplementary material for: Methane Emission by Camelids
Source: PLoS One. 2014 Apr 9;9(4):e94363. doi: 10.1371/journal.pone.0094363 (PMC3981797; doi:10.1371/journal.pone.0094363)
Supplement: Table S1 — Dataset including the literature data used for statistical comparison of camelids and ruminants. (DOCX) [file pone.0094363.s001.docx]

**Methane emission by camelids – Supporting Information**

Marie T. Dittmann, Ullrich Runge, Richard Lang, Dario Moser, Cordula Galeffi, Michael Kreuzer, Marcus Clauss

**Table S1. Dataset including the literature data used for statistical comparison of camelids and ruminants.**

| **Species** | **Source** | **BM** | **CH_4_** | | | |
| --- | --- | --- | --- | --- | --- | --- |
|  |  | **kg** | **l d^-1^** | **l kg^-1^ DMI** | **l kg^-1^ dNDF** | **% DEI** |
| **Camelids** |  |  |  |  |  |  |
| *Vicugna pacos* | [1] * | 42.0 | 2.5 | 4.2 | 20.2 | 1.6 |
| *Vicugna pacos* | [2] * | 48.0 | 2.9 | 5.7 | 24.6 |  |
| *Vicugna pacos* | [3] * | 63.3 | 20.8 | 24.7 | 130.9 |  |
| *Vicugna pacos* | [4] | 64.0 | 22.4 | 22.4 |  |  |
| *Lama glama* | [5] * | 64.0 | 51.6 | 32.1 | 77.1 |  |
| *Lama glama* | [6] | 100.8 | 33.7 | 28.5 | 81.1 | 12.6 |
| *Camelus dromedarius* | [7] * | 330.0 | 69.7 | 15.9 |  |  |
| **Ruminants** |  |  |  |  |  |  |
| *Capra hircus* | [8] | 32.5 | 17.2 | 19.0 |  | 8.9 |
| *Capra hircus* | [9] | 33.3 | 22.9 | 23.1 |  | 8.6 |
| *Capra hircus* | [10] | 33.5 | 26.2 | 40.4 |  | 13.3 |
| *Capra hircus* | [11] | 37.3 | 16.3 | 21.4 |  | 7.9 |
| *Capra hircus* | [12] | 40.0 | 14.8 | 22.1 |  |  |
| *Capra hircus* | [13] | 60.0 | 40.0 |  |  | 13.2 |
| *Capra hircus* | [13] | 100.0 | 67.0 |  |  | 13.2 |
| *Ovis aries* | [14] | 26.2 | 19.5 | 31.1 | 82.8 | 13.8 |
| *Ovis aries* | [13] | 27.0 | 23.0 |  |  | 11.0 |
| *Ovis aries* | [15] | 32.4 | 21.6 | 27.3 |  |  |
| *Ovis aries* | [16] | 33.7 | 25.1 | 26.1 | 77.3 | 7.5 |
| *Ovis aries* | [13] | 37.0 | 34.0 |  |  | 12.0 |
| *Ovis aries* | [17] | 38.6 | 25.7 | 29.8 |  |  |
| *Ovis aries* | [18] | 39.9 | 21.5 |  |  |  |
| *Ovis aries* | [18] | 40.0 | 15.1 |  |  |  |
| *Ovis aries* | [19] | 40.3 | 24.4 | 31.8 |  |  |
| *Ovis aries* | [18] | 42.2 | 14.5 |  |  |  |
| *Ovis aries* | [20] | 43.0 | 18.0 | 18.8 |  |  |
| *Ovis aries* | [21] | 43.6 | 22.3 | 24.3 |  |  |
| *Ovis aries* | [18] | 44.0 | 23.6 |  |  |  |
| *Ovis aries* | [22] | 45.3 | 34.2 | 31.1 |  |  |
| *Ovis aries* | [19] | 46.9 | 27.1 | 29.3 |  |  |
| *Ovis aries* | [18] | 47.2 | 27.5 |  |  |  |
| *Ovis aries* | [19] | 48.2 | 23.9 | 22.5 |  |  |
| *Ovis aries* | [18] | 58.1 | 32.5 |  |  |  |
| *Ovis aries* | [18] | 58.7 | 12.4 |  |  |  |
| *Ovis aries* | [23] | 58.8 | 50.6 | 31.6 |  | 11.9 |
| *Ovis aries* | [19] | 59.2 | 29.0 | 23.1 |  |  |
| *Ovis aries* | [21] | 59.7 | 24.2 | 27.1 |  |  |
| *Ovis aries* | [13] | 60.0 | 36.0 |  |  | 13.2 |

**Table S1. continued**.

| **Species** | **Source** | **BM** | **CH_4_** | | | |
| --- | --- | --- | --- | --- | --- | --- |
|  |  | **kg** | **l d^-1^** | **l kg^-1^ DMI** | **l kg^-1^ dNDF** | **% DEI** |
| *Ovis aries* | [6] | 65.1 | 33.4 | 28.9 | 84.8 | 13.4 |
| *Ovis aries* | [21] | 67.4 | 27.2 | 25.2 |  |  |
| *Ovis aries* | [21] | 72.6 | 28.9 | 22.4 |  |  |
| *Ovis aries* | [24] | 90.6 | 28.1 | 34.4 | 106.8 | 15.8 |
| *Ovis aries* | [24] | 92.7 | 33.7 | 24.8 | 76.3 | 10.0 |
| *Ovis aries* | [24] | 98.7 | 29.1 | 21.6 | 93.0 | 11.0 |
| *Ovis aries* | [13] | 110.0 | 57.0 |  |  | 13.2 |
| *Bos indicus* | [25] | 358.5 | 258.4 | 48.5 | 103.3 | 20.0 |
| *Bos taurus* | [26] | 272.0 | 155.4 | 32.0 |  | 11.2 |
| *Bos taurus* | [26] | 302.0 | 166.6 | 30.5 |  | 10.6 |
| *Bos taurus* | [27] | 545.5 | 415.0 | 27.6 | 56.2 | 7.7 |
| *Bos taurus* | [28] | 606.0 | 354.2 | 24.8 |  |  |
| *Bos taurus* | [28] | 606.0 | 385.5 | 27.1 |  |  |
| *Bos taurus* | [28] | 606.0 | 435.8 | 29.1 |  |  |
| *Bos taurus* | [28] | 606.0 | 469.2 | 31.7 |  |  |
| *Bos taurus* | [18] | 610.0 | 279.3 |  |  |  |
| *Bos taurus* | [29] | 611.0 | 451.2 | 33.4 | 59.2 | 10.6 |
| *Bos taurus* | [30] | 649.0 | 472.1 | 35.0 | 65.6 | 12.1 |

Values are ordered by species and body mass. Empty cells indicate that the corresponding data were not available from that source. Note that in many cases not all available data were taken from the respective publication, but only those measurements that were obtained with roughage-only diets. Values indicated with asterisks were not included in the analysis as animals were not kept on a roughage-only diet or methane measurements were not carried out by respirometry. BM body mass; DMI dry matter intake; dNDF digestible neutral detergent fiber; DEI digestible energy intake.

**References**

1. Liu Q, Dong CS, Li HQ, Yang WZ, Jiang JB, et al. (2009) Effects of feeding sorghum-sudan, alfalfa hay and fresh alfalfa with concentrate on intake, first compartment stomach characteristics, digestibility, nitrogen balance and energy metabolism in alpacas (*Lama pacos*) at low altitude. Livest Sci 126: 21-27.

2. Liu Q, Dong CS, Li HQ, Yang WZ, Jiang JB, et al. (2009) Forestomach fermentation characteristics and diet digestibility in alpacas (*Lama pacos*) and sheep (*Ovis aries*) fed two forage diets. Anim Feed Sci Technol 154: 151-159.

3. Pinares-Patiño CS, Ulyatt MJ, Waghorn GC, Lassey KR, Barry TN, et al. (2003) Methane emission by alpaca and sheep fed on lucerne hay or grazed on pastures of perennial ryegrass/white clover or birdsfoot trefoil. J Agric Sci 140: 215-226.

4. Pinares-Patiño CS, Franco FE, Battistotti M, Molano G, Sandoval E, et al. (2013) Methane emissions from alpaca and sheep fed lucerne hay as either chaff or pellets. Adv Anim Biosci 4: 584 (Abstr.).

5. Carmean BR, Johnson KA, Johnson DE, Johnson LW (1992) Maintenance energy requirement of llamas. Am J Vet Res 53: 1696-1698.

6. Vernet J, Vermorel M, Jouany JP (1997) Digestibility and energy utilisation of three diets by llamas and sheep. Ann Zootech 46: 127-137.

7. Guerouali A, Laabouri FZ (2013) Estimates of methane emission from the camel (*Camelius dromedarius*) compared to dairy cattle (*Bos taurus*). Adv Anim Biosci 4: 286 (Abstr.).

8. Puchala R, Animut G, Patra A, Detweiler GD, Wells JE, et al. (2012) Methane emissions by goats consuming Sericea lespedeza at different feeding frequencies. Anim Feed Sci Technol 175: 76-84.

9. Islam M, Abe H, Hayashi Y, Terada F (2000) Effects of feeding Italian ryegrass with corn on rumen environment, nutrient digestibility, methane emission, and energy and nitrogen utilization at two intake levels by goats. Small Rumin Res 38: 165-174.

10. Animut G, Puchala R, Goetsch AL, Patra AK, Sahlu T, et al. (2008) Methane emission by goats consuming diets with different levels of condensed tannins from lespedeza. Anim Feed Sci Technol 144: 212-227.

11. Puchala R, Animut G, Patra AK, Detweiler GD, Wells JE, et al. (2012) Effects of different fresh-cut forages and their hays on feed intake, digestibility, heat production, and ruminal methane emission by Boer × Spanish goats. J Anim Sci 90: 2754-2762.

12. Puchala R, Min BR, Goetsch AL, Sahlu T (2005) The effect of a condensed tannin-containing forage on methane emission by goats. J Anim Sci 83: 182-186.

13. Vermorel M (1997) Emissions annuelles de méthane d'origine digestive par les ovins, les caprins et les équins en France. INRA Prod Anim 10: 153-161.

14. Tiemann TT, Lascano CE, Wettstein H, Mayer AC, Kreuzer M, et al. (2008) Effect of the tropical tannin-rich shrub legumes Calliandra calothyrsus and Flemingia macrophylla on methane emission and nitrogen and energy balance in growing lambs. Animal 2: 790-799.

15. Sun XZ, Pacheco D, Molano G, Luo DW (2013) Sheep fed fresh forage rape (*Brassica napus subsp. Oleifera L*.) have lower methane emissions compared with perennial ryegrass (*Lolium perenne L.*). Adv Anim Biosci 4: 271 (Abstr.).

16. Carulla JE, Kreuzer M, Machmüller A, Hess HD (2005) Supplementation of *Acacia mearnsii* tannins decreases methanogenesis and urinary nitrogen in forage-fed sheep. Aus J Agric Res 56: 961-970.

17. Nolan JV, Hegarty RS, Hegarty J, Godwin IR, Woodgate R (2010) Effects of dietary nitrate on fermentation, methane production and digesta kinetics in sheep. Anim Prod Sci 50: 801-806.

18. Ritzman EG, Benedict FG (1938) Nutritional physiology of the adult ruminant. Washington DC, USA: Carnegie Institute.

19. Archimede H, Rira M, Eugene M, Morgavi DP, Anais C, et al. (2013) Intake, total-tract digestibility and methane emission of Texel and Blackbelly sheep fed C4 and C3 grasses tested simultaneously in a temperate and a tropical area. Adv Anim Biosci 4: 285 (Abstr.).

20. Zhao G, Li B, Wang M, Zhang T, Zheng W (2013) The rumen methane production of rice straw ammoniated with different levels of urea, evaluated using in vitro incubation. Adv Anim Biosci 4: 296 (Abstr.).

21. Fraser MD, Fleming H, Theobald V, Sanderson R, Moorby JM (2013) Effect of body size on feed intake and methane emissions from ewes offered fresh forage. Adv Anim Biosci 4: 358 (Abstr.).

22. Hammond KJ, Hoskin SO, Burke JL, Waghorn GC, Koolaard JP, et al. (2011) Effects of feeding fresh white clover (*Trifolium repens*) or perennial ryegrass (*Lolium perenne*) on enteric methane emissions from sheep. Anim Feed Sci Technol 166: 398-404.

23. Margan DE, Graham NM, Minson DJ, Searle TW (1988) Energy and protein values of four forages, including a comparison between tropical and temperate species. Anim Prod Sci 28: 729-736.

24. Franz R, Soliva CR, Kreuzer M, Steuer P, Hummel J, et al. (2010) Methane production in relation to body mass of ruminants and equids. Evol Ecol Res 12: 727-738.

25. Kurihara M, Magner T, Hunter RA, McCrabb GJ (1999) Methane production and energy partition of cattle in the tropics. Br J Nutr 81: 227-234.

26. Hironaka R, Mathison GW, Kerrigan BK, Vlach I (1996) The effect of pelleting of alfalfa hay on methane production and digestibility by steers. Sci Total Environ 180: 221-227.

27. Staerfl SM, Amelchanka SL, Kälber T, Soliva CR, Kreuzer M, Zeitz JO (2012) Effect of feeding dried high-sugar ryegrass (‚AberMagic’) on methane and urinary nitrogen emissions of primiparous cows. Livest Sci 150: 293-301.

28. Podesta SC, Hatew B, Klop G, Van Laar H, Kinley RD, et al. (2013) The effect of nitrogen fertilization level and stage of maturity of grass herbage on methane emission in lactating cows. Adv Anim Biosci 4: 272 (Abstr.).

29. Hindrichsen IK, Wettstein HR, Machmüller A, Kreuzer M (2006) Methane emission, nutrient degradation and nitrogen turnover in dairy cows and their slurry at different milk production scenarios with and without concentrate supplementation. Agric Ecosyst Environ 113: 150-161.

30. Klevenhusen F, Kreuzer M, Soliva CR (2011) Enteric and manure-derived methane and nitrogen emissions as well as metabolic energy losses in cows fed balanced diets based on maize, barley or grass hay. Animal 5: 450-461.
